# Supplementary material for: Towards unified AI-driven fracture mechanics: the extended deep energy method (XDEM)
Source: Nat Commun. 2026 Aug 15;17:8492. doi: 10.1038/s41467-026-76748-1 (PMC13484471; doi:10.1038/s41467-026-76748-1)
Supplement: Supplementary file 3 [file 41467_2026_76748_MOESM3_ESM.zip › pic/Results/Crack_propagation/Kinking/shear_contourf.pdf]

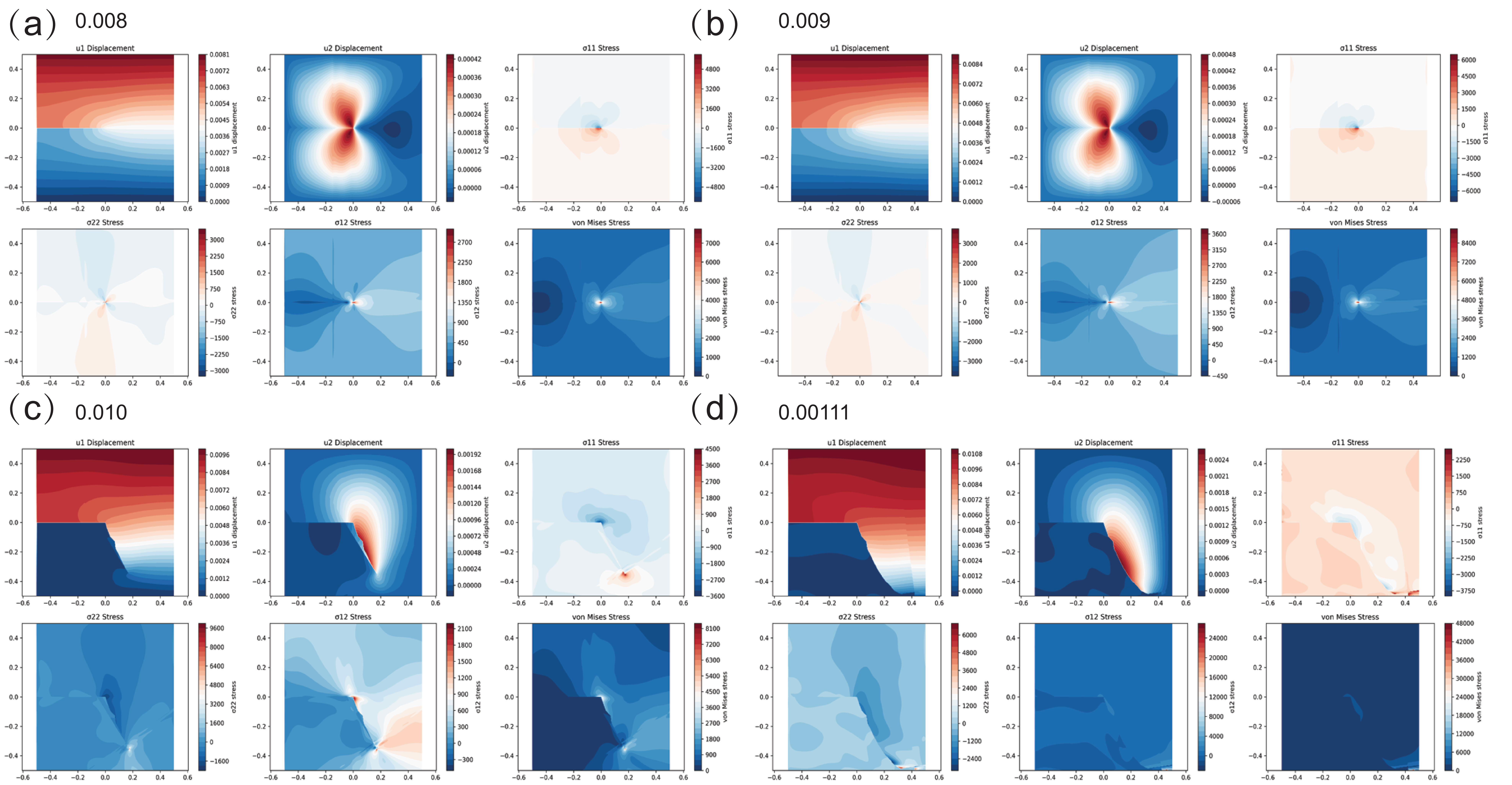

(a) 0.008
(b) 0.009
(c) 0.010
(d) 0.00111
u1 Displacement
u2 Displacement
σ11 Stress
σ22 Stress
σ12 Stress
von Mises Stress
u1 displacement
u2 displacement
σ11 stress
σ22 stress
σ12 stress
von Mises stress
